# Killing effects of Huaier Granule combined with DC-CIK on nude mice transplanted with colon carcinoma cell line

**DOI:** 10.18632/oncotarget.17687

**Published:** 2017-05-08

**Authors:** Wen-Wen Sun, Jin-Xia Dou, Lin Zhang, Li-Kui Qiao, Na Shen, Qiang Zhao, Wen-Yuan Gao

**Affiliations:** ^1^ Tianjin Key Laboratory for Modern Drug Delivery & High-Efficiency, School of Pharmaceutical Science and Technology, Tianjin University, Tianjin 300072, China; ^2^ Institute of Fundamental Research, Tianjin Academy of Traditional Chinese Medicine Affiliated Hospital, Tianjin 300120, China; ^3^ Department of Center Laboratory, Tianjin 4th Center Hospital, Tianjin 300140, China; ^4^ Department of Pathology, Tianjin 4th Center Hospital, Tianjin 300140, China; ^5^ Department of Massage Manipulation, Tianjin Academy of Traditional Chinese Medicine Affiliated Hospital, Tianjin 300120, China

**Keywords:** DC-CIK, Huaier Graunle, HT-29 colon carcinoma cell line, *in vivo* killing experiment

## Abstract

This study aims to compare the efficacy of different treatments for nude mice transplanted with HT-29 colon carcinoma cell line. BalB/C nude mice were transplanted with HT-29 colon carcinoma cell line and randomly divided into four groups, with 5 mice in each group: blank control group, DC-CIK group, Huaier Granule group, and Huaier Granule group combined with DC-CIK group (combined treatment group). For DC-CIK group and combined treatment group, 1×10^6^ DC-CIK cells were injected via the tail vein 4 days after transplantation. The injection was performed twice weekly for a total of 2 weeks. For Huaier Granule group and combined treatment group, Huaier Granule was administered at the dose of 20 g/60 g, by dissolving 20 g of Huaier granules in 600 ml of pure water. Intragastric administration of 0.2 ml of granules was performed once daily for 3 weeks. For the blank control group, equal volume of normal saline was given. Tumor size and body weight of nude mice were measured every 2 days during the 3-week treatment. The mice were sacrificed at the end of treatment to harvest tumors. Key genes of the signaling pathway were detected by RT-PCR. At the end of treatment, mice in combined treatment group, DC-CIK group and Huaier Granule group remained stable emotionally with normal mobility and water and food intake. However, in the blank control group, the mobility was restricted starting from the third week and the mice were on the verge of dying. The expression of PI3KR1, Akt, Wnt1, CTTNB1, Notch1, Notch2 and Notch3 genes were all downregulated significantly in the combined treatment group compared with DC-CIK group and Huaier Granule group (P<0.05). Therefore, the combined treatment of Huaier Granule combined with DC-CIK achieved the best effect in nude mice transplanted with HT-29 colon carcinoma cell line.

## INTRODUCTION

Colon carcinoma is a common gastrointestinal cancer in clinic. In China, it ranks the 3^rd^ and 5^th^ position in terms of incidence and mortality, respectively. China has witnessed an increasing prevalence of colon carcinoma since 1980s, by 38.56% in 15 years [1]. Because of atypical early symptoms, colon carcinoma is usually associated with a high misdiagnosis rate, high malignancy, delayed diagnosis and easy metastasis. The five-year survival of early-stage colon carcinoma is about 90% [2], in contrast to 7% for advanced colon carcinoma [3].

At present, an integrated treatment consisting of biotherapy and traditional Chinese medicine is gaining popularity for cancers. Constant efforts are made to find out the reason for the low 5-year survival rate in colon carcinoma. Steward first proposed the concept of tumor stem cell (TSC) in 1994, which was considered as the root cause of tumor development, metastasis, recurrence and resistance to chemotherapy and radiotherapy due to its infinite self-renewal and differentiation potential. Since then, many studies were devoted to TSC and to develop tumor immunotherapy. Dendritic cells (DCs) are the most powerful antigen-presenting cells that can activate initial T cells and specific immune response and facilitate non-specific cytotoxic killing. They are important antigen carriers in anti-tumor immunity. Cytokine-induced killer cells (CIK) are potent killers of tumor cells. With rapid *in vitro* proliferation and broad-spectrum killing effects, CIK cells are sensitive to drug-resistant tumor cells and considered ideal immunocompetent cells.

Huaier is a type of medicinal fungus that contains over 18 amino acids and different minerals. It can be used to enhance immunity and inhibit tumor cell growth [5]. Colon carcinoma was induced in nude mice by transplanting with HT-29 colon carcinoma cell line. Huaier Granule was combined with DC-CIK to treat tumor in nude mice and the treatment efficacy was observed. To discuss the mechanism, expressions of key genes in the signaling pathway were detected.

## RESULTS

### Tumor-bearing nude mice

**Figure 1:**
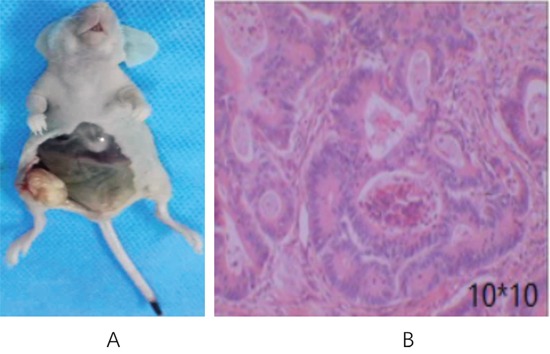
Transplanted tumor in nude mice inoculated with CD133^+^ HT29 colon carcinoma cells **(A)** Gross anatomy; **(B)** HE staining of tumor tissues (100× magnification).

Nodules were formed at 4-5 d after inoculation of 1×10^5^ CD133^+^ HT29 colon carcinoma cells into the nude mice. The transplanted tumors grew rapidly with clear margins and hard texture. The tumors were harvested and covered with yellow envelop (Figure 1A). Pathological examination of the tumor tissues indicated that it was moderately differentiated colon adenocarcinoma (Figure 1B).

### Morphology and immunophenotyping of DC-CIK cells

**Figure 2:**
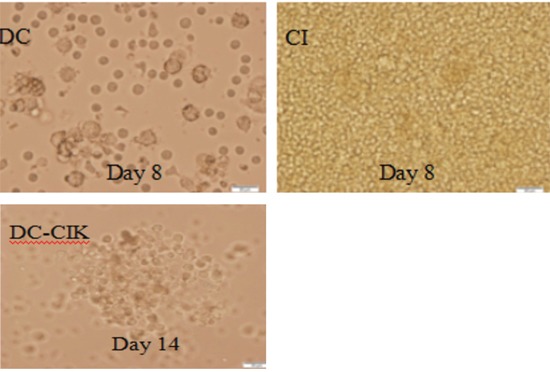
Morphology of DC-CIK cells under the light microscope (magnification 400×)

DC progenitors were harvested 2 hours after adherence of peripheral blood mononuclear cells to the wall. The adherent cells were round. At 5d of culture, dendrites were seen on the cell surface (Figure 2). At the early stage of culture, CIK cells were round and the morphology was uniform. They were co-cultured with DCs at 8d. As more cells proliferated over time, the cell morphology became irregular and large suspending colonies appeared (Figure 2).

**Figure 3:**
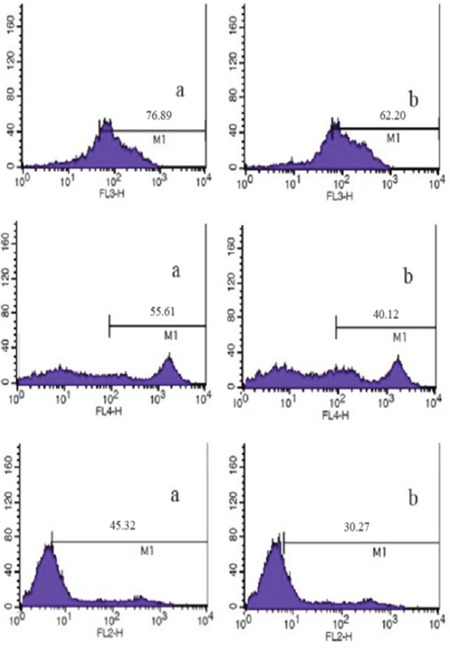
Analysis of CD3^+^ DC-CIK (a) and CIK (b) cells by flow cytometry

**Figure 4:**
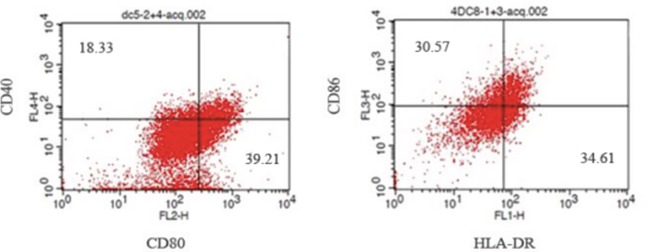
Analysis of DCs cells positive for CD40, CD80, CD86 and HLA-DR by flow cytometry

According to flow cytometry, after co-culture of DCs and homologous CIK cells, the number of CD3^+^CD8^+^ and CD3^+^CD56^+^ CIK cells increased (Figure 3; Table 1) (P<0.05). At 8d, the percentage (5) of DCs positive for CD40, CD80, CD86 and HLA-DR was 18.33±2.12, 39.21±3.81, 30.57±2.56 and 34.61±3.21, respectively (Figure 4).

**Table 1:** Immunophenotyping of CIK cells (n=10, %, $\bar{x}\pm s$)

Group	CD3^+^	CD3^+^CD8^+^	CD3^+^CD56^+^
DC-CIK group (d14)	76.89±7.31*	55.61±6.21*	45.32±6.11*
CIK group (d8)	62.20±7.11	40.12±6.10	30.27±5.45

*P<0.05 for intergroup comparison.

### Comparison of survival of tumor-bearing nude mice

**Figure 5:**
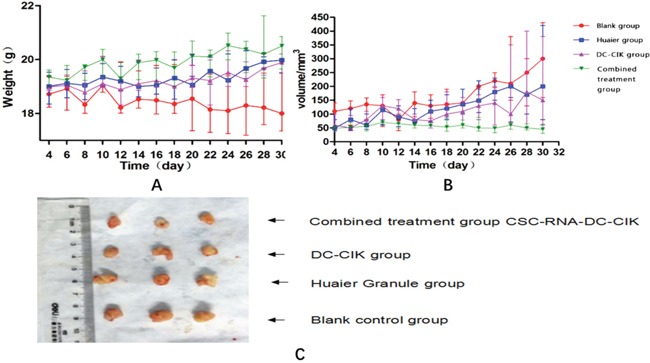
Physical examinations of tumor-bearing nude mice during treatment **(A)** Curve of body weight; **(B)** Curve of tumor size in each group during treatment; **(C)** Tumor size of each group after treatment.

Nude mice in combined treatment group, DC-CIK group and Huaier Granule group remained emotionally stable throughout the course of treatment. In contrast, the nude mice in blank control group were dispirited with reduction in body weight. Mobility was restricted starting from the third week and the nude mice were on the verge of dying. At the end of treatment, nude mice in combined treatment group, DC-CIL group and Huaier Granule group gained weight steadily, whereas the body weight of nude mice in the blank control group declined slightly (Figure 5A and 5B).

### Comparison of tumor growth

Tumor size did not increase significantly for combined treatment group, DC-CIK group and Huaier Granule group during the course of treatment. As to the rate of tumor growth, combined treatment group<DC-CIK group<Huaier Granule group. However, after treatment, tumor size and weight of combined treatment group <DC-CIK group< Huaier Granule group<blank control group (Figure 5C).

### Influence of different treatments on expressions of key genes

**Figure 6:**
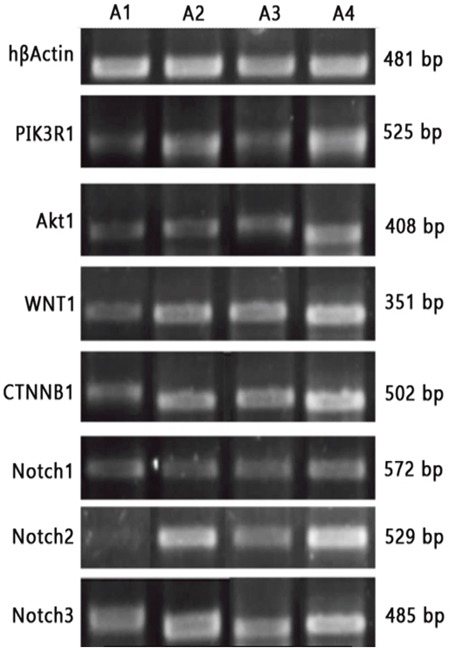
RT-PCR detections of mRNA expressions of key genes A1: DC-CIK-Huaier Granule group; A2: DC-CIK group; A3: Huaier Granule group; A4: Blank control group.

After treatment, there were significant differences in mRNA expressions of PI3KR1 and Akt, Wnt1, CTNNB1, Notch 1, Notch2 and Notch 3 (Table 2) for combined treatment group, DC-CIK group and Huaier Granule group as compared with the blank control group (P<0.05) (Figure 6). The differences in mRNA expressions of the above genes reached a significant level for combined treatment group as compared with DC-CIK group and Huaier Granule group (P<0.05). However, the difference between DC-CIK group and Huaier Granule group did not reach a significant level (P>0.05).

**Table 2:** Integral optical density of key genes (n=5, OD value, $\bar{x}\pm s$)

Group	PI3KR1		Akt1
Blank control group (1)	100±18.79		93±15.23
Huaier Granule group (2)	51±10.23		35±5.64
DC-CIK group (3)	49±10.19		28±4.28
Combined treatment group (4)	36±6.78		21±4.01
*F*	26.0503**		79.9289**
**Group**	**Wnt1**		**CTNNB1**
Blank control group (1)	54±11.74		114±20.37
Huaier Granule group (2)	20±4.98		72±15.32
DC-CIK group (3)	19±4.36		63±14.29
Combined treatment group (4)	8±3.09		26±4.32
*F*	41.5216**		29.9425**
**Group**	**Notch1**	**Notch2**	**Notch3**
Blank control group (1)	52±10.97	84±14.76	110±22.69
Huaier Granule group (2)	37±8.23	24±5.01	35±5.72
DC-CIK group (3)	36±8.03	26±5.12	30±5.01
Combined treatment group (4)	-	23±4.39	12±3.49
*F*	89.2065**	10.3497**	64.1406**

^*^P<0.05; P<0.05 for any other groups except P>0.05 for group (2) vs. group (3).

## DISCUSSION

Tumor stem cells are considered the root cause of tumor recurrence and drug resistance. Existing treatments for cancers mainly target the proliferating tumor cells, but not the tumor stem cells. Although the tumors disappear in the short term after treatment, the surviving tumor stem cells can form tumor afterwards. Therefore, selective killing of tumor stem cells may provide a radical cure for cancers [4].

DC, the most important antigen-presenting cell, can recognize and present antigens specific to tumor stem cells. So DC can be used for targeted therapy of cancers. Provenge^®^ immunotherapy utilizing DC has achieved desired effect against melanoma in clinic [6]. CIK cells are immune effector cells, predominantly CD3^+^CD56^+^T cells, produced by PBMC under the stimuli from many cytokines (CD3McAb, IL-2, IFN-γ and IL-1α) [7]. Zhou et al. [8] induced DCs, CIK cells and LAK cells as effector cells using human mononuclear cells, which were then applied to *in vitro* killing test on colon carcinoma SW480 cells. Result showed that the killing effect of DC-CIK cells on colon carcinoma SW480 cells was stronger than LAK-DC, CIK or LAK cells alone.

Huaier Granule contains over 18 amino acids and a diversity of minerals. It has been now used to treat cancers by enhancing immunity and inhibiting cancer growth [5, 9].

Huaier Granule combined with DC-CIK cells was applied to tumor-bearing nude mice transplanted with HT-29 colon carcinoma cells. *In vivo* killing test showed that the nude mice in the combined treatment group, DC-CIL group and Huaier Granule group remained emotionally stable with normal water and food intake and no restriction to mobility. This indicated that immunotherapy and Huaier Granule had no adverse or toxic effects.

The development, proliferation and differentiation of tumor stem cells are related to several signaling pathways, such as Wnt/β-catenin, Wnt, Notch, HOX and Bmi-1 pathways [9, 10]. As compared with the blank control group, mRNA expression of PI3KR1, Akt, Wnt1, CTNNB1, Notch1, Notch2 and Notch3 were downregulated significantly in combined treatment group, DC-CIK group and Huaier Granule group. As compared with DC-CIK group and Huaier Granule group, the combined treatment group also showed a significant downregulation of the above genes.

To conclude, combined treatment is more powerful in killing tumor stem cells than either DC-CIK or Huaier Granule alone. This sheds new light onto the treatment of colon carcinoma.

## MATERIALS AND METHODS

### Materials

HT-29 colon carcinoma cell line was donated by Tianjin Medical University Cancer Institute and Hospital. DMEM was purchased from Gibco (USA). Triton X-100 was purchased from Amresco (USA). EasyPure RNA Kit ER101 was purchased from Transgen (USA). KOD plus was purchased from Toyobo (USA). (sc-7382) Bcl-2 (C-2) antibody was purchased from SANTA CRUZ (USA). Alexa Fluor^®^ 488 Goat Anti-Mouse IgG was purchased from Invitrogen (USA). One Step TUNEL Apoptosis Assay Kit (#C1088) was purchased from Beyotime (Jiangsu, China). Matrigel matrix was purchased from Corning (USA). PCR primers were synthesized by Beijing Aok-real Detection Technology Development Co., Ltd. RNA extraction kit was purchased from Qiagen (USA). Reverse transcription kit was purchased from Takara (Japan). 0.25% trypsin was purchased from Beijing Solarbio Science & Technology Co., Ltd. CD133 antibody-coated magnetic bead was purchased from Miltenyi (Germany). Huaier Granule was purchased from Qidong Gaitianli Pharmaceutical Co., Ltd.

### Animals

Twenty SPF male Balb/c nude mice aged 3-4 weeks and weighing 16-18 g were purchased from Vital River Laboratory Animal Technology Co., Ltd. The mice were bred in IVC cages (laboratory animal license: 11400700106716).

### Equipment

IVC cages (Suzhou Suhang Technology Equipment Co., Ltd.), biosafety cabinet (Thermo, USA), magnetic bead separator (Miltenyi, Germany), microscope (Olympus, Japan), electrophoresis apparatus (Bio-Rad, USA), flow cytometer (BD, USA), automated cell counter (Countstar, USA), inverted fluorescence microscope (Zeiss, Germany), PCR instrument (ABI, USA), and gel imaging system (DNR, Israel).

### Cell culture

HT29 colon carcinoma cells were grown in DMEM and cultured at 37°C in a humidified incubator (5% CO2/95% air). Half of the culture medium was replaced every 2 days, and cell passage was performed when the cells grew to 80% confluency.

### Magnetic bead separation

Log-phase cells were harvested and 1×10^8^ cells were resuspended in 300 *μl* of PBS. The cells were incubated with 100 *μl* of Fc receptor and 100 μl of CD133 antibody at 4°C for 30 min. Then CD133^+^ cells were collected.

### Preparation and identification of CIK cells

Culture of CIK cells: From healthy subject 50 ml of white blood cells was collected. Non-adherent cells were harvested and added into 1640 medium containing 10% FBS and 50 ng/mL IFN-γ. Then 500 U/ml IL-2, 5 ng/ml IL-1α and 50 ng/ml CD3 antibody were added 24 h later and half of the culture medium was replaced every 2-3 days. CIK cells and DC-CIK cells were collected at 8d and 14d, respectively, and counted. Cells positive for CD3, CD56 and CD8 antibodies were analyzed on a flow cytometer.

### Preparation and identification of DCs

Culture of DCs: From healthy subject 50 ml of white blood cells was collected. Non-adherent cells were harvested and grown in 1640 medium containing 10% FBS, IL-4 and 1 000 U/ml GM-CSF. DCs were harvested at 8d of culture and those positive for CD40, CD80, HLA-DR and CD86 antibodies were detected using a flow cytometer.

### Transplantation of HT-29 colon carcinoma cells into nude mice

Twenty Balb/c nude mice were acclimatized for 1 week. The above-mentioned HT29 colon carcinoma cells positive for CD133^+^ were adjusted to the concentration of 1×10^5^/ml. The cells were mixed evenly with serum-free medium and matrigel matrix at 1:1 proportion. Using a microinjector, 0.2 ml of the cells was inoculated to the right axilla of nude mice. Tumor formation was observed every 2 days. Appearance of hard nodules in the axilla indicated that the tumor was successfully induced.

### HE staining

Tumors were harvested and cut into about 2 mm thickness along the maximum diameter. Tissues were fixed in neutral formalin, embedded in paraffin, sliced and subjected to HE staining.

### Immunotherapy

Tumor-bearing nude mice were divided into 4 groups, with 5 mice in each group: (1) blank control group, 0.2 ml of normal saline was injected via the tail vein twice weekly; (2) DC-CIK group, 0.2 ml of DC-CIK cells (1×10^6^) was injected via the tail vein twice weekly; (3) Huaier Granule group, 20 g of Huair Granule was dissolved in 600 ml of pure water and 0.2 ml of the solution was given by gastric irrigation at the dose of 20 g/60 kg once daily; (4) combined treatment group, 0.2 m of DC-CIK cells (1×10^6^) was injected via the tail vein twice weekly besides gastric irrigation of 0.2 ml of the above-mentioned Huaier Granule once daily. The treatment lasted for 3 weeks for the four groups. The time of the first immunotherapy was 4 days after inoculation of HT29 colon carcinoma cells. Tumor size was measured every 2 days during the course of 3 weeks. Maximum diameter (length) and two minimum diameters (width) of the tumor were measured with a vernier caliper and tumor volume (mm^3^)=length × width × width ×0.52.

### RT-PCR detection of key genes related to the signaling pathway

20 mg of transplanted tumor tissues cryopreserved at -80°C was added with liquid nitrogen, ground well and transferred to an RNase-free EP tube. Total RNA was extracted using Qiagen Rneasy mini kit. cDNA was synthesized from total RNA using TAKARA PrimeScript RT-PCR Kit. With hβActin gene as internal control, primers were designed for the key genes and synthesized by Genewiz. The genes detected were as follows (in agreement with the gene names from NCBI): PI3K/Akt: PI3KR1, Akt1; Wnt/β-catenin: Wnt1, CTNNB1; Notch: Notch1, Notch2, Notch3. (Table 3).

**Table 3:** Primer sequences

Genes to be amplified	Primer No.	Primer sequence
**PIK3R1**	PI3KR1-2-F	ACGTTTTGGCTGACGCTTTC
	PI3KR1-2-R	AGGTCCCGTCTGCTGTATCT
		**Product length: 525**
**Akt1**	Akt-5-NB	TACGAGATGATGTGCGGTCG
	Akt-3-NB	CGAGTAGGAGAACTGGGGGA
		**Product length: 408**
**WNT1**	Wn1t-1-F	TGTGAACGTAGCCTCCTCCA
	Wnt1-1-R	TCACACGTGCAGGATTCGAT
		**Product length: 351**
**CTNNB1**	CTNNB1-2-F	ATGATGGTCTGCCAAGTGGG
	CTNNB1-2-R	GCAGCTGCACAAACAATGGA
		**Product length: 502**
**Notch1**	Notch1-2-F	GCAGAGGCGTGGCAGACTA
	Notch1-2-R	CCCGTTCTTGCAGTTGTTTCC
		**Product length: 572**
**Notch2**	Notch2-5	TGAGTAGGCTCCATCCAGTC
	Notch2-3	TGGTGTCAGGTAGGGATGCT
		**Product length: 529**
**Notch3**	Notch3-5	TCTTGCTGCTGGTCATTCTC
	Notch3-3	TGCCTCATCCTCTTCAGTTG
		**Product length: 485**
**ACTB**	hbActin-s	CGGGAAATCGTGCGTGACATTA
	hbActin-a	CGGACTCGTCATACTCCTGCTTG
		**Product length: 481**

### Statistical analysis

SPSS 10.0 software was used for statistical analysis. Measurement data were expressed as mean ± standard deviation ($\bar{x}\pm s$). To compare the means within and across the groups, ANOVA were performed. For further intergroup comparisons, Bonferroni correction was used. P<0.05 was considered significant difference.
